# Targeting the CXCR4/CXCL12 Axis in Cancer Therapy: Analysis of Recent Advances in the Development of Potential Anticancer Agents

**DOI:** 10.3390/molecules30061380

**Published:** 2025-03-20

**Authors:** Gerardina Smaldone, Francesca Di Matteo, Roberta Castelluccio, Valeria Napolitano, Maria Rosaria Miranda, Michele Manfra, Pietro Campiglia, Vincenzo Vestuto

**Affiliations:** 1 Department of Pharmacy, University of Salerno, Via G. Paolo II, 84084 Fisciano, Italy; gsmaldone@unisa.it (G.S.); fdimatteo@unisa.it (F.D.M.); rcastelluccio@unisa.it (R.C.); vnapolitano@unisa.it (V.N.); pcampiglia@unisa.it (P.C.); 2 PhD Program in Drug Discovery and Development, University of Salerno, 84084 Fisciano, Italy; mmiranda@unisa.it; 3 Department of Health Science, University of Basilicata, Viale dell’Ateneo Lucano 10, 85100 Potenza, Italy; michele.manfra@unibas.it

**Keywords:** cancer, CXCR4/CXCL12 axis, small molecules, antitumor drugs

## Abstract

Cancer, a leading cause of premature death, arises from genetic and epigenetic mutations that transform normal cells into tumor cells, enabling them to proliferate, evade cell death, and stimulate angiogenesis. Recent evidence indicates that chemokines are essential in tumor development, activating receptors that promote proliferation, invasion, and metastasis. The CXCR4/CXCL12 signaling pathway is gaining attention as a promising target for cancer therapy. CXCR4, a chemokine receptor, is often overexpressed in various types of cancer, including kidney, lung, brain, prostate, breast, pancreas, ovarian, and melanomas. When it binds to its endogenous ligand, CXCL12, it promotes cell survival, proliferation, and migration, crucial mechanisms for the retention of hematopoietic stem cells in the bone marrow and the movement of lymphocytes. The extensive expression of CXCR4 in cancer, coupled with the constant presence of CXCL12 in various organs, drives the activation of this axis, which in turn facilitates angiogenesis, tumor progression, and metastasis. Given the detrimental role of the CXCR4/CXCL12 axis, the search for drugs acting selectively against this protein represents an open challenge. This review aims to summarize the recent advancements in the design and development of CXCR4 antagonists as potential anticancer agents.

## 1. Introduction

Cancer is one of the leading causes of premature death worldwide and certainly represents a significant challenge for the world of scientific research [1]. The development of the tumor state is linked to the accumulation of genetic and epigenetic mutations that cause normal cells to acquire the ability to proliferate uncontrollably, resist cell death, and induce angiogenesis [2]. The formation of metastases is the primary problem in cancer treatment and is the leading cause of death in cancer patients. It is characterized by a series of stages that represent a ‘metastatic cascade’: during the process of metastatization, cancer cells leave the primary site, circulate in the blood stream, adapt to the new cellular environment at a secondary site, and escape the deadly fight with immune cells [3]. Tumor recurrence and the lack of effective treatment options contribute to high mortality rates; therefore, comprehending the mechanisms underlying tumorigenesis and progression is crucial for unraveling the intricate biology of cancer.

Chemokines are chemotactic cytokines that control the migration of cells between tissues and the positioning and interactions of cells within tissue [4]. Several scientific studies suggest that chemokines can directly modulate tumor growth by inducing cancer cell proliferation and preventing apoptosis. They may also indirectly modulate tumor growth through their effects on tumor stromal cells by inducing the release of growth and angiogenic factors from cells in the tumor microenvironment.

CXCL12, also known as CXC motif chemokine 12, is a chemokine, which is a small, secreted peptide mainly known for immune cell recruitment. Chemokines in general are also involved in other pathological conditions including inflammation, atherosclerosis, hematopoiesis, and cancer [5]. CXCL12, known also as stromal cell-derived factor-1 (SDF-1), is a homeostatic CXC chemokine featuring seven isoforms (α, β, γ, δ, ε, θ, and the predicted isoform iso7), each of which play distinct roles in various biological contexts. The α subtype is the most prevalent, contributing to myoblast migration during myogenesis and muscle regeneration, regulating hematopoietic stem cell populations in the bone marrow, and facilitating neuromodulation within the central nervous system [6]. Subtype β exhibits enhanced pro-angiogenic properties and is predominantly found in highly vascularized organs, such as the kidneys, liver, and spleen [7]. Meanwhile, in human [8] and mice [9], the γ subtype is greatly expressed in organs such as the heart and brain [10]. This subtype has a higher affinity for its receptor, leading to prolonged downstream effects and a significant role in the metastatic process of breast cancer. CXCL12 is the natural binder of both CXCR4 and CXCR7, and its biological activity is mediated by the high-affinity interaction with these receptors.

**Figure 1:**
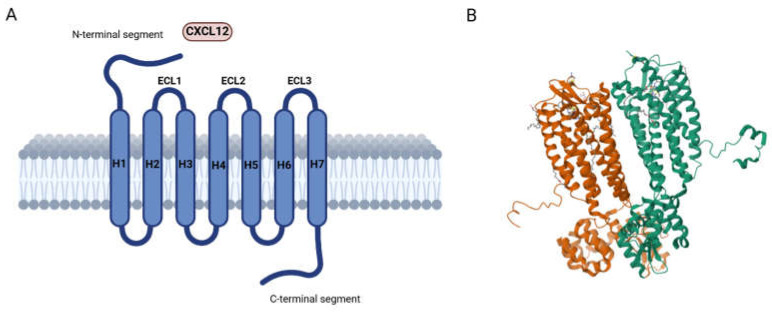
(**A**) Schematic diagram of CXCR4; (**B**) 2.5 A structure of the CXCR4 chemokine receptor in complex with small-molecule antagonist IT1t (Protein Data Bank identifier 3ODU [14]).

C-X-C chemokine receptor type 4 (CXCR4) (Figure 1), initially named LESTR (leukocyte-derived seven-transmembrane domain receptor), is widely expressed on hematopoietic cells, including CD34^+^ HSCs, T lymphocytes, B lymphocytes, monocytes and macrophages, neutrophils, and eosinophils, as well as in brain, lung, colon, heart, kidney, and liver endothelial and epithelial cells, microglia, astrocytes, neuronal cells, and progenitor cells, including endothelial and smooth muscle progenitors [11]. It belongs to the family of chemokine receptors, commonly classified into CR, CCR, CXCR, and CX3CR receptors based on the type of chemokine they bind. These receptors are differentially expressed on different cell types. CXCR4 is a highly conserved seven-transmembrane-spanning G-protein-coupled receptor consisting of 352 amino acid residues, including an amino (N) terminus, three extracellular and intracellular loops, seven TM helices, and a carboxyl (C) terminus [12]. Helix VII consists of long helical turns and allows the generation of a disulfide bond between Cys274 and Cys28 in the N-terminal region. This is essential for the binding of CXCL12 [13], which, to the best of our knowledge, is the only confirmed chemokine that binds to CXCR4.

CXCR7 is another G-protein-coupled receptor (GPCR), also known as atypical chemokine receptor 3 (ACKR3). This receptor is activated by CXCL11 and CXCL12. CXCL11 is induced by interferon-γ in human microvascular endothelial cells (HMEC-1), hepatocytes, and hepatic stellate cells in liver inflammation, while CXCL12 is involved in stem cell survival, proliferation, and homing. In some studies, CXCR7 has been considered a scavenger receptor for CXCL12 because it removes the chemokine from the circulation by binding and sequestering it [15], while other studies suggest that CXCR7 induces intracellular signals associated with CXCR4, for instance, the activation of the ERK (extracellular signal-regulated kinase) and AKT (protein kinase B) pathways, receptor internalization, cell survival, proliferation, and adhesion [16].

## 2. CXCR4: Localization and Physio-Pathological Role

**Figure 2:**
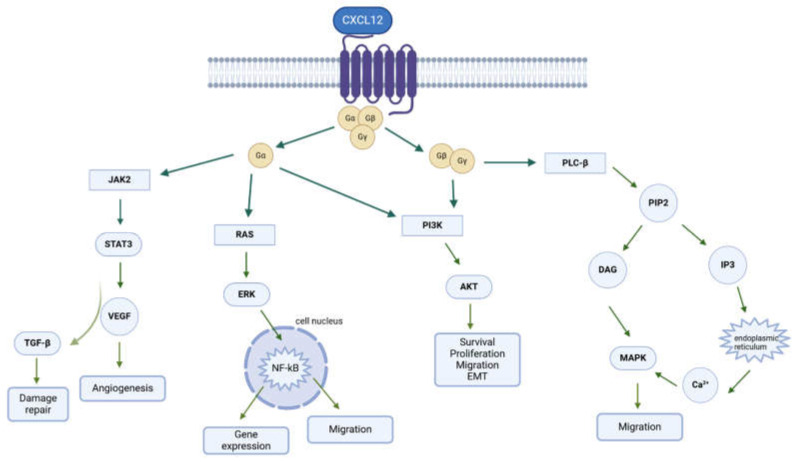
The signaling pathways in the CXCR4/CXCL12 biological axis.

CXCR4 belongs to the superfamily of G-protein-coupled receptors (GPCRs), and it is coupled with the Gα_i_ subunit (Figure 2). The Gα_i_ subunit inhibits adenylyl cyclase (AC) and activates mitogen-activated protein kinases (MAPKs) and the phosphatidylinositol-3-kinase (PI3K) pathway. The Gβγ subunit promotes the activation of phospholipase C (PLC), which causes the hydrolysis of phosphatidylinositol 4,5-bisphosphate (PIP2) to diacylglycerol (DAG) and inositol triphosphate (IP3), leading to the release of Ca^2+^ from intracellular stores and contributing to cell migration [17]. Activated PI3K also promotes the triggering of the AKT pathway, which plays a key role in tumor cell survival, given its capability in inactivating the BAD (BCL2-associated agonist of cell death) protein and GSK3β, as well as stabilizing β-catenin with the promotion of gene transcription and proliferation. The latter pathway drives the phosphorylation of many focal adhesion components and contributes to the reorganization of the actin cytoskeleton, resulting in the changes necessary for cell migration [13,18]. Furthermore, it has been reported that activated MAPKs translocate into the nucleus and phosphorylate the nuclear protein ELK-1, leading to proliferation and differentiation in different cell types [19].

The Gα subunit also actives the Ras and Rac/Rho pathways, with a consequent phosphorylation of ERK and P38, which are involved in cell apoptosis, cell-cycle progression, and proliferation [20]. Furthermore, CXCR4 is involved in the activation of the JAK/STAT pathway, which contributes to cell morphological changes such as polarization, resulting in chemotactic responses. After ligand binding, the JAK2 and JAK3 kinases are activated and phosphorylate the CXCR4 receptor, with a subsequent recruitment of members of the transcription factor family STAT [21]. The intracellular mechanisms transmitted by the CXCR4 pathway are switched off by the phosphorylation catalyzed by GRK, inducing arrestin 2/3 recruitment and thus leading to the subsequent internalization of the receptor. In addition, CXCR4 ubiquitination by AIP4 on the plasma membrane results in its transport and degradation in lysosomes. However, a portion of the internalized receptor can also be recycled and returned to the plasma membrane [22].

Primarily, the complex CXCR4/CXCL12 controls the regeneration of organs and tissues such as the lung, heart, liver, and nervous system, and plays a critical role in the development of the brain, heart, vascular system, hematopoietic system, and germ cells [23,24]. In fact, CXCL12 is responsible for the retention and homing of hematopoietic progenitor and stem cells in the bone marrow microenvironment [25]. Moreover, CXCR4 plays a pivotal role in the nervous system. Following injury, CXCL12 binding to CXCR4, expressed at the tip of the motor axon, promotes the structural and functional recovery of muscle junctions in the peripheral nervous system [26]. Instead, in the CNS, CXCR4 is expressed on neuronal progenitor cells, while CXCL12 is released by astrocytes. In the CNS, cells’ regenerative capacity is limited because the activation of the receptor by CXCL12 does not cause proliferation, but only induces the differentiation of human embryonic stem cells into neuronal stem cells [27].

Despite the pivotal role of CXCR4/CXCL12 in homeostasis, this axis may also be associated with pathological states, and in particular with tissue damage, such as heart infarct, limb ischemia, toxic liver damage, excessive bleeding, and total body irradiation, as well as superficial tissue injury, blisters, and desquamation caused by chemotherapy drugs [28].

In addition, the CXCR4 receptor is overexpressed in more than 23 different cancers, including kidney, lung, brain, prostate, breast, pancreas, ovarian, and melanomas, and contributes to tumor growth, angiogenesis, metastasis, and therapeutic resistance [28,29].

## 3. CXCR4/CXCL12 Axis in Cancer

The upregulation of CXCR4 expression in malignant cells depends on several mechanisms. In particular, among the well-known inducers of CXCR4 expression, there is VEGF, which in turn is induced upstream by HIF-1, a heterodimeric transcription factor that responds to decreases in tissue oxygen concentrations and is therefore responsible for CXCR4 upregulation. Therefore, in expanding tumors characterized by a high presence of hypoxic regions, CXCR4 levels are significantly higher than in non-tumor cells, facilitating survival and escape from the primary tumor mass; indeed, in addition to facilitating distant metastasis, HIF-1 has been shown to induce CXCR4 in gliomas, leading to increased proliferation, resistance to apoptosis, and local invasion [30].

The CXR4/CXL12 axis is also involved in the regulation of metastasis; indeed, chemokine receptors may intervene in each of the crucial steps of the metastasis mechanism to facilitate tumor progression, including tumor cell adhesion to the endothelium, extravasation from the blood vessels, metastatic colonization, angiogenesis, proliferation, and protection from host response through the activation of key survival pathways, such as ERK/MAPK, PI-3K/AKT/mTOR, or JAK/STAT. Furthermore, the important role of chemokines in facilitating communication between tumor cells and non-neoplastic cells in the tumor microenvironment (TME) has been widely recognized. These non-neoplastic cells include endothelial cells and fibroblasts, and promote the infiltration and activation of tumor-associated neutrophils and macrophages (TAMs) within the TME [31].

**Proliferation**. The mechanism of tumor proliferation is a multi-step process involving several factors and pathways, including the CXCL12/CXCR4 axis. In particular, the CXCR4/CXCL12/PI3K/AKT signaling pathway has been recognized as an important prognostic marker for the progression of adamantinomatous craniopharyngiomas (adaCP) and a potential indicator of recurrence. Indeed, it has been shown that the binding of CXCL12 to its receptor CXCR4 significantly increases both the expression of β-catenin and its translocation into the nucleus via the PI3K/AKT signaling pathway, thereby stimulating the transcription of target genes: this process promotes the proliferation and migration of adaCP cells, facilitating the progression of the disease [32]. The involvement of CXCR4/CXCL12 in the growth and metastasizing mechanism of breast cancer through the activation of the JAK2/STAT3 pathway has also been demonstrated, highlighting how the simultaneous expression of CXCR4 and p-STAT3 at the tissue level results in a link to tumor size, lymph node metastasis, and histological tumor grade [33]. Klein et al. demonstrated how the overexpression of CXCR4 or stimulation with CXCL12 promoted proliferation and tumor growth in neuroblastoma cells through the activation of the MAPK signaling pathway; furthermore, the inhibition of CXCR4 with the high-affinity CXCR4 antagonist BL-8040 prevented tumor growth and reduced tumor cell survival [34]. The activation of the MAPK signaling pathway, mediated by CXCR4 overexpression, was also found to be responsible for the increase in precancerous lesions of pancreatic duct cancer, increasing the frequency and intensity of their appearance [35].

**Angiogenesis**. There is much evidence linking the CXCR4 axis with angiogenesis; for example, the lack of CXCR4 or CXCL12 in mice leads to aberrant large vessel formation and abnormal vessel development. In addition, endothelial cells of large vessels in the tumor stroma also overexpressed the CXCR4 receptor, suggesting that the CXCR4/CXCL12 axis may be highly involved in tumor angiogenesis. There are four mechanisms by which this effect is actuated [36]:

(i) The upregulation of vascular endothelial growth factor (VEGF) expression in tumor tissue;
(ii) Reductions in the expression of glycolytic enzyme phosphoglycerate kinase 1 (PGK1), which in turn suppresses the secretion of VEGF;
(iii) The upregulation of several angiogenesis-associated genes in cancer cells;
(iv) Routing the recruitment of endothelial progenitor cells to the vicinity of new vessels.

Among all these factors, the role of VEGF remains the most important. First, the CXCR4/CXCL12 axis induces AKT phosphorylation, which leads to an upregulation of VEGF [37]. In addition, the presence of VEGF and HIF-1 stimulates the expression of CXCR4 in human brain microvascular endothelial cells, and this event promotes angiogenesis in human glioblastoma [30]. CXCL12 promotes the upregulation of angiogenesis-associated genes, such as IL-6, by the phosphorylation of ERK and the activation of the NF-kB complex [38].

CXCR4^+^ proangiogenic cells include immature and mature hematopoietic cells, endothelial precursor cells, and smooth muscle cell progenitors, which have direct or indirect proangiogenic properties. CXCL12 plays a role in the mobilization and recruitment of these cells to the neoangiogenic niches supporting the revascularization of ischemic tissue and tumor growth.

**Metastasis**. The CXCR4/CXCL12 axis is heavily connected to cancer metastasis, as it is involved in several steps: adhesion, invasion, cell proliferation, and survival. In fact, CXCL12 expression levels are increased at sites of metastasis such as the brain, bone marrow, lung, and liver, so cancer cells can exploit the CXCR4/CXCL12 axis to establish distant organ metastasis. On the other hand, it has been reported that the downregulation of CXCR4 by natural products suppresses the migration and invasion of some cancers, such as liver cancer, confirming the latter hypothesis [39].

The overexpression of the CXCR4/CXCL12 axis induces metastasis formation in different ways: in the tumor microenvironment (TME), hypoxia and toxins cause an increase in CXCL12 levels, generating the migration of CXCR4-expressing tumor cells. When CXCL12 activates its receptor, angiogenesis, tumor cell survival, proliferation, and chemoresistance are promoted. This cytokine primarily regulates tumor cell adhesion through laminin (a component of the basement membrane), fibrinogen, stromal cells, and endothelial cells [40]. Studies in prostate cancer models show that the exposure of PC3 cells, derived from bone metastases, to increasing concentrations of CXCL12 leads to an up-regulation of the CXCR4 gene, and this mechanism is mediated by NF-kB: this results in increased adhesion (175–200%) and the trans-endothelium migration of cancer cells. In other tumor lines, this increased adhesion of tumor cells to the endothelium has, instead, been correlated with the induction of integrin expression, as they mediate the binding and response of cells to the extracellular matrix [40,41].

Epithelial-to-mesenchymal transition (EMT) is a key process in the development of metastasis. CXCR4/CXCL12 axis activation stimulates the SHH (Sonic hedgehog) signaling pathway, which is associated with EMT and the loss of cell adhesion [42]. Moreover, CXCR4/CXCL12 signaling upregulates survival via the MED/ERK and PI3K/AKT pathways, giving rise to cell-cycle progression and EMT in multiple tumors, for example, in human sacral chondrosarcoma [43], in glioblastoma [44], and in hepatocellular carcinoma [45]. In colorectal cancer, EMT and invasion are stimulated by the CXCR4/CXCL12 axis through the Wnt/β-catenin signaling pathway [46].

Both CXCR4 and CXCL12 promote the production of metalloproteases. For example, CXCL12 promotes the invasion of bone by myeloma cells by stimulating MMP-9 and MT1-MMP expression [47].

A hypoxic environment is common in solid cancer, and, in this environment, endothelial cells upregulate HIF-1, which in turn promotes the upregulation of CXCL12. For this reason, CXCR4^+^ cancer cells are attracted to peripheral blood vessels [48]. Also, CXCL12 acts as a recruiter of CXCR4^+^ cancer cells, and the physiological expression of CXCL12 in organs such as liver, lungs, and bone marrow promotes metastasis growth in these sites by recruiting CXCR4^+^ cancer cells. CXCR4^+^ cancer cells easily migrate where CXCL12 is highly expressed, towards organs such as the lungs, lymph nodes, and bones, which represent the most common metastasis sites [49].

**Tumor microenvironment.** The role of the microenvironment in tumor progression is crucial. The tumor microenvironment (TME) consists of several non-cancerous cells, such as stromal fibroblasts, endothelial cells, and immune cells; proteolytic enzymes; growth factors; inflammatory cytokines; and the extracellular matrix (ECM) [50]. CXCL12 plays a key role in regulating the interaction between the tumor and the TME. The interaction between the TME and the tumor stimulates proliferation, survival, angiogenesis, and metastasis of the tumor itself, and all these pathways are in turn promoted by CXCL12. The latter is secreted by carcinoma-associated fibroblasts (CAFs); it interacts with CXCR4 expressed on cancer cells, and the activation of the axis stimulates cancer proliferation directly [51].

Beider et al. observed that CXCL12 secreted by both myeloma multiple cells and bone marrow cells regulates monocyte migration; monocyte differentiation into macrophages not only promotes cancer proliferation, but also supports the immunosuppressive microenvironment around the tumor [52].

Again, the CXCR4/CXCL12 axis promotes chemo-resistance, and chemotherapy can upregulate CXCR4/CXCL12 expression in multiple forms of cancer.

## 4. Small Molecules Targeting the CXCR4/CXCL12 Axis

**Figure 3:**
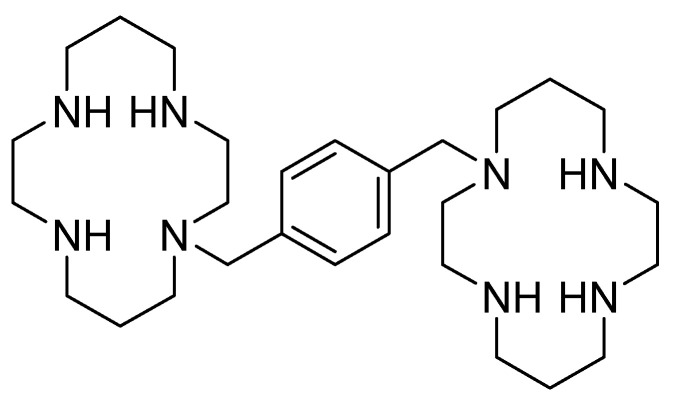
AMD3100 structure.

Considering the increasing relevance of the CXCR4/CXCL12 axis in cancer, several effective antagonists have been discovered in the last few years, most of them using AMD3100 as a lead compound. The structure of AMD3100 is shown in Figure 3. It contains a byciclam scaffold: molecular modelling suggests that one cyclam ring of AMD3100 interacts with Asp171 in TM-IV, whereas the other ring is sandwiched between the carboxylic acid groups of Asp262 and Glu288 from TM-VI and -VII, respectively [53].

Plerixafor (AMD3100) is a macrocyclic compound that acts as an irreversible antagonist against the binding of CXCR4 with its ligand CXCL12 [54]. Plerixafor mobilizes hematopoietic stem and progenitor cells for transplantation better than granulocyte colony-stimulating factor (G-CSF) alone. It also increases T-cell trafficking in the blood and spleen, as well as the CNS. Plerixafor is currently used in combination with G-CSF as a hematopoietic stem cell mobilizer [55]: blocking CXCR4 receptors with AMD3100 demonstrated the prevention of tumor recurrence in orthotopic xenografts of glioblastoma in a mouse model [56].

Moreover, AMD3100 is receiving interest as an anti-cancer agent that disrupts the CXCR4/CXCL12 chemokine receptor interaction between neoplastic cells and their microenvironment in tumor progression and metastasis. In several studies, AMD3100 has been shown to inhibit tumor cell proliferation and migration, promoting tumor cell apoptosis in vitro and slow tumor growth in vivo. The antitumor effect of AMD3100 has been associated with a reduced activation of ERK 1/2 and AKT, which are downstream signaling pathways of CXCR4 [57,58,59].

### 4.1. AMD3100 Derivatives

**Figure 4:**
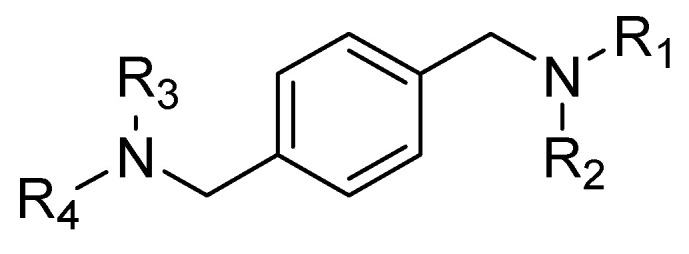
General structure of AMD3100 derivatives.

In 2007, Zhan et al. [60] used AMD3100 as a lead compound to design a small library of derivatives as potential CXCR4 antagonists. The compounds contained the general structure reported in Figure 4, retaining the same structural features as AMD3100. The basic nitrogen centers that replace the cyclam moieties carry out the same interactions as acidic amino acids, but eliminate the possible coordination of the cyclam rings with metal ions, which is probably the cause of the cardiotoxicity of AMD3100 [61].

All the synthesized compounds were tested via a competitive binding assay, using TN14003, a potent CXCR4 antagonist, as a reference (IC_50_ = 0.6 nM) [61].

**Figure 5:**
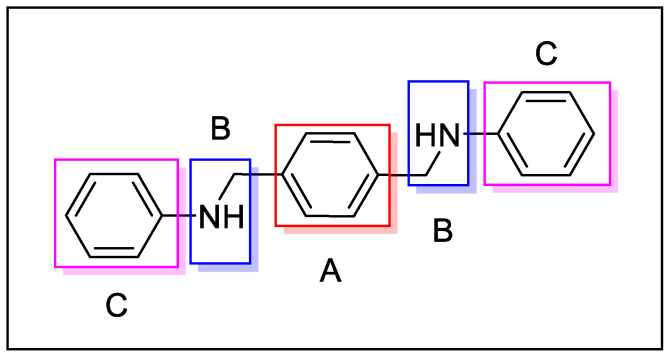
The structure of derivative 15, showing sections subjected to synthetic modifications.

Derivative 15 was found to be the most active of the series. This compound was used as a lead to better understand the structure–activity relationship of CXCR4 antagonists; in particular, three sections were subjected to synthetic modification: the central scaffold, the linker, and the terminal aromatic ring (Figure 5).

Following this approach, several considerations were made:

1. The central aromatic ring is critical for high CXCR4 affinity. The substitution of the central phenyl ring (Figure 5, A) with a cyclohexane ring leads to a complete loss of activity. In the same way, the removal of the central ring yields unactive compounds. In addition, the replacement of the central ring with a bicycle or tricycle ring decreases the activity, as well as the ortho-substitution of sections B and C (Figure 5).
2. One carbon separation between the central phenyl ring and the nitrogen of the acyclic linker is essential for high potency. The insertion of a methyl group on benzyl carbon or nitrogen leads to a significant decrease of in. Moreover, the elongation of the aliphatic chain between segments A and C leads to a loss of activity.
3. Anti-CXCR4 activity is much more sensitive to para substitution on the terminal aromatic rings compared to meta and ortho substitution; in particular, electron-donating groups in the para positions of the terminal phenyl rings (Figure 5, C) increase the activity. On the other hand, the introduction of electron-withdrawing groups leads to a reduction in activity [60].

**Figure 6:**
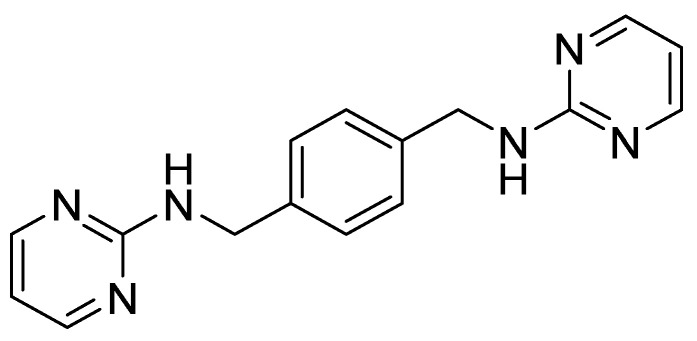
MSX-122 structure.

MSX-122 (Figure 6) was one of the most promising molecules obtained by this SAR study; MSX-122 is a potent CXCR4 antagonist, and it has been demonstrated to inhibit metastasis and inflammation by several in vitro and in vivo studies. Matrigel invasion and cAMP modulation assays were performed to assess the potency of the compound, while the in vivo assays were performed on three different tumor animal models, including breast, lung, and head/neck cancers [62].

Unfortunately, MSX122 has been withdrawn from clinical trials due to its toxicity (ID NCT00591682).

### 4.2. Amide and Sulfonamide Derivatives

**Figure 7:**
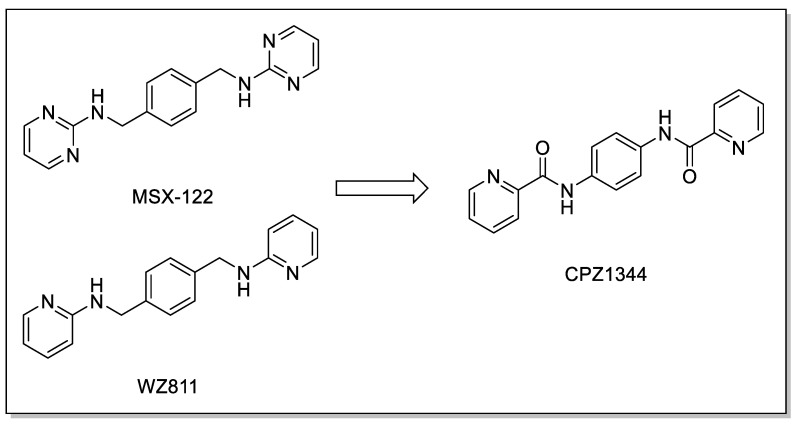
Structures of the CXCR4 inhibitors MSX-122 and WZ811, and the newly developed CXCR4 inhibitor CPZ1344.

Recently, the replacement of amine with an amide scaffold led to a novel picolinamide-based CXCR4/CXCL12 axis inhibitor, CPZ1344 [63]. It was designed from a combination of the structural information derived from WZ811, another potent CXCR4 inhibitor, and MSX-122 [60], based on the overlapping structural features of AMD3100, replacing the key N-macrocyclic regions that mediate CXCR4 binding (Figure 7). CPZ1344 activity against the CXCR4/CXCL12 axis was investigated on glioblastoma cells. Glioma cells overexpressing CXCR4 were treated with increasing doses of the compound, resulting in the inhibition of cell growth in a dose-dependent manner, especially in the U87 cell line, and in increase in apoptosis without apparent toxicity. These findings demonstrated the anticancer effects of CPZ1344 and its potential as a novel anti-GBM therapeutic [64].

**Figure 8:**
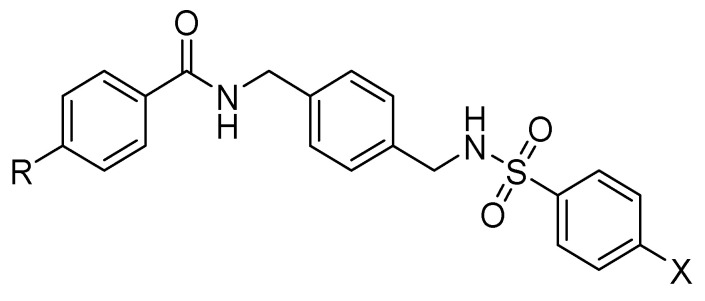
Insertion of amidic or sulfonamidic moieties on p-xylylene derivatives.

In more recent years, Bai et al. found through computer-aided drug design screening that the introduction of amidic and sulfonamidic moieties not only maintains the interaction with the CXCR4 receptor, but also blocks the invasion of CXCR4-positive tumor cells (Figure 8). In this study, structurally related p-xylylene derivatives containing a sulfonamide moiety were identified, and most of them showed an excellent binding affinity to CXCR4, confirming the amide–sulfamide pharmacophore as a suitable substitution for the development of CXCR4 antagonists [65].

When an X group is replaced by a strong withdrawing group, such as a nitro group, the interaction with CXCR4 increases, as demonstrated by the competitive binding assay (IC_50_ = 100 nM). Moreover, the introduction of weak withdrawing groups (-F or -Cl) also results in a high affinity towards the CXCR4 receptor. On the other hand, the introduction of electron-donating groups showed non-regular behavior in terms of increasing or decreasing affinity [66].

On the benzamide side, the replacement of R- with methyl, methoxy, or chloride leads to an increased potency in the Matrigel assay. However, the substitution with fluorine in position 3 or 4 of the benzenamide group demonstrates a better effect.

**Figure 9:**
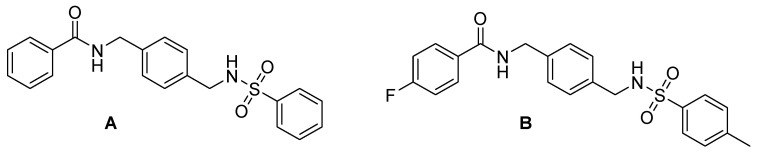
Novel amide/sulfonamide-based derivatives as CXCR4 antagonists.

To evaluate the in vivo anti-inflammatory effects of the amide–sulfonamide derivatives, a xylene-induced ear edema experiment was performed: the compounds reported in Figure 9 exhibited the best suppressive activity, with 62% (Figure 9A) and 75% (Figure 9B) inhibition, respectively [67].

### 4.3. Tetrahydroquinoline–Benzimidazole-Based Scaffold

**Figure 10:**
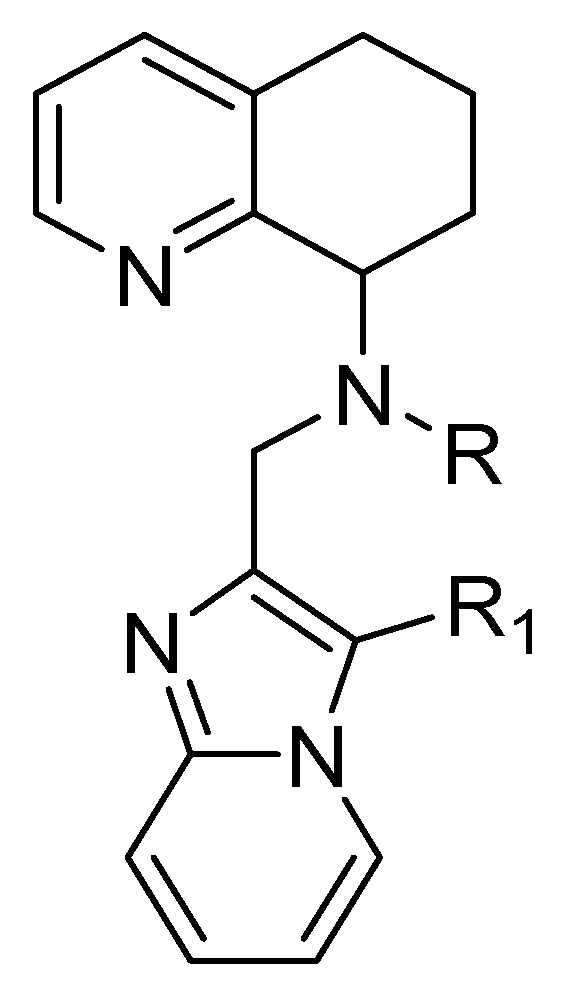
General structure of tetrahydroquinoline–benzimidazole-based compounds.

The byciclam rings of AMD3100 were also replaced with a tetrahydroquinoline–benzimidazole-based scaffold to maintain the same basic features necessary to interact with the CXCR4 receptor. The general structure of these derivatives is shown in Figure 10; the R group might be replaced by an alkyl or hetero-alkyl chain, while the N-rich-containing side chain should replace the R1 group. As previously reported, basic features are essential to bind acidic amino acids, in particular Asp171 and Asp262, located in the transmembrane regions of TM-IV and TM-VI.

**Figure 11:**
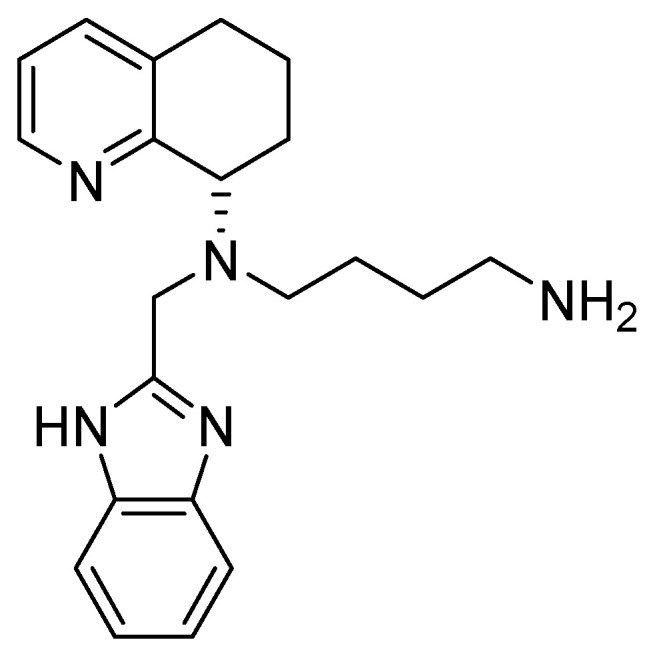
AMD11070 structure.

These efforts led to the discovery of AMD11070, which improved the oral bioavailability with respect to the previous derivatives and reduced the side effects linked to the byciclam scaffold (Figure 11).

The main structural modifications that have proven to be effective as a replacement for the cyclam nucleus are the following:

A chiral tetrahydroquinoline (THQ);
A basic heterocycle;
A butyl amine chain [68].

In April 2024, AMD11070 was approved by the FDA under name of Xolremdi (mavorixafor) in patients 12 years of age and older with WHIM syndrome (warts, hypogammaglobulinemia, infections, and myelokathexis) to increase the number of circulating mature neutrophils and lymphocytes. WHIM syndrome is a rare genetic disease that causes the body’s immune system to not function properly, reducing the number of mature neutrophils and lymphocytes. The effectiveness of Xolremdi was evaluated in a 52-week, randomized, double-blind, placebo-controlled trial that enrolled 31 adolescents and adults with WHIM syndrome (NCT03995108).

However, AMD11070 has also been demonstrated to be effective in melanoma; in fact, both AMD3100 and AMD11070 significantly blocked the chemotaxis of CHL-1 cells (melanoma human cell line) towards CXCL12, but AMD11070 showed to be more effective at inhibiting the migration of the melanoma cell line A375 than AMD3100 (78% vs. 21% inhibition, respectively) [69].

**Figure 12:**
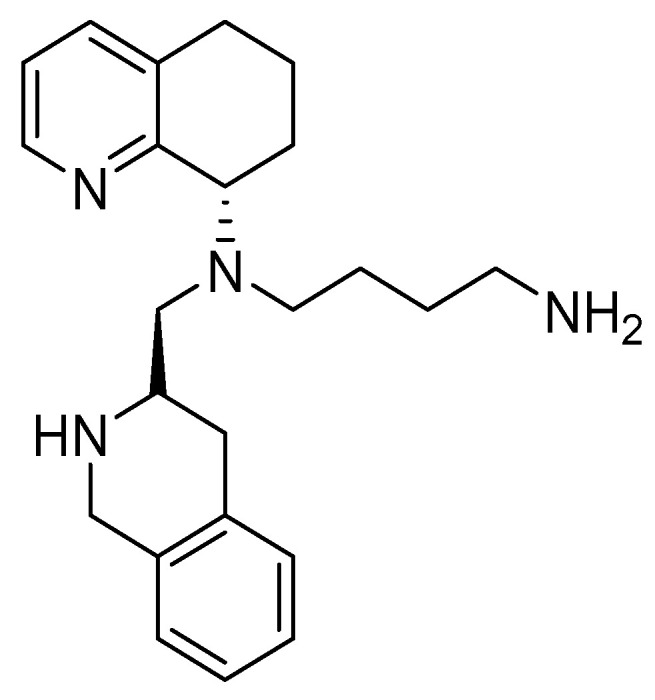
TIQ-15 structure.

TIQ-15 (Figure 12) is a mavorixafor analogue that was found to inhibit CXCL12-induced calcium flux/release in Chem-1 cells, confirming that the tetrahydroquinoline scaffold is suitable for the development of anticancer activity, blocking the CXCR4 pathway [70].

### 4.4. Indole Scaffold Derivatives

**Figure 13:**
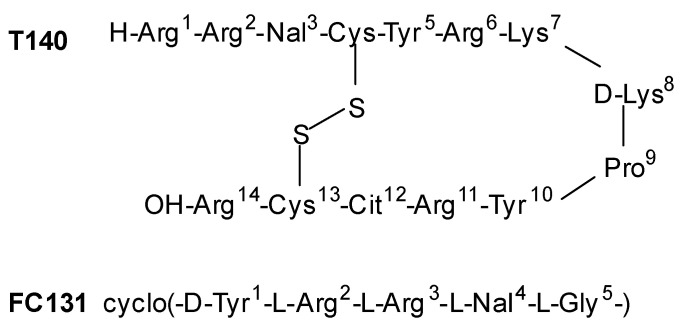
The peptides used as lead compounds to develop indole-based CXCR4 antagonists. Nal, *L*-3-(2-naphthyl)alanine; Cit, *L*-citrulline. Adapted from Ueda et al. [70,71].

Another class of CXCR4 antagonists endowed with an indole scaffold was developed, starting from the structures of the peptide antagonists T140 (IC_50_ = 2.5 nM) and peptide FC131 shown in Figure 13.

The SAR studies on peptide FC131, derived from T140, showed that three functional groups are necessary for interaction with CXCR4: an aromatic ring such as 2-naphthyl- or 3-indolyl group at position 4; a guanidine moiety at position 3; and a guanidine group at position 2 or a phenol group at position 1 [70].

**Figure 14:**
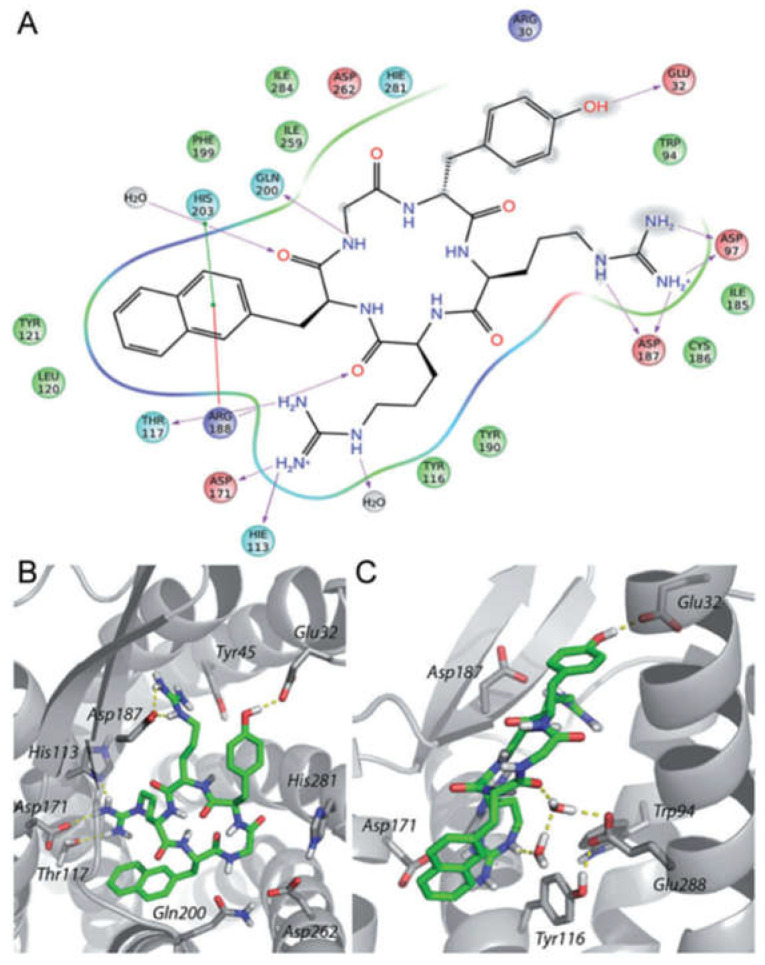
Binding mode of peptide FC131 with CXCR4. (**A**) Two-dimensional representation of the FC131-binding site in CXCR4. Three-dimensional model of FC131 binding in CXCR4, as seen from the top (**B**) or the side (**C**). Adapted from Thiele et al. [71].

In fact, molecular docking studies (Figure 14) revealed the central role in the interaction with CXCR4 of the following amino acid residues of FC131: D-Tyr^1^ is directed towards Glu32 at the N-terminal end of the receptor; Arg^2^ interacts with Asp187 and Asp97; Arg^3^ interacts with His113/Asp171; and finally, the aromatic portion of Nal^4^ is positioned between Arg188 and His203, establishing cation–π and π-π interactions, respectively [71].

**Figure 15:**
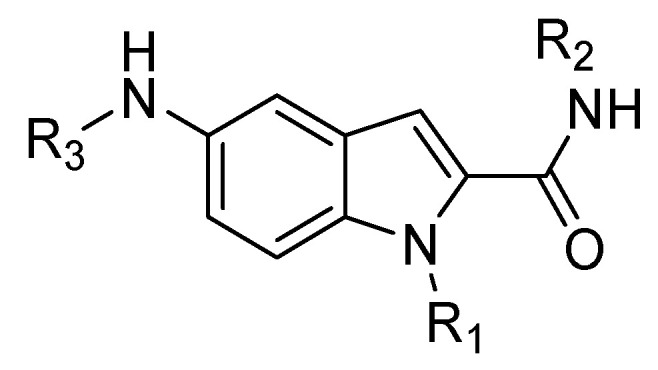
General structure of the indole-based CXCR4 antagonists developed.

Based on these studies, the indole nucleus was chosen as the central core for the synthesis of novel potential CXCR4 antagonists. 5-Aminoindole-2-carboxylic acid was selected as a starting material, given its structural features: the possibility to easily introduce three substituents at positions 1, 2, and 5 (Figure 15); the molecular modelling information that suggested that indole is able to maintain the spatial requirements of the parental peptide; and the well-known importance of the indole scaffold as a privileged structure in medicinal chemistry, being widely investigated for the development of both anticancer and antiviral small molecules [72,73].

**Figure 16:**
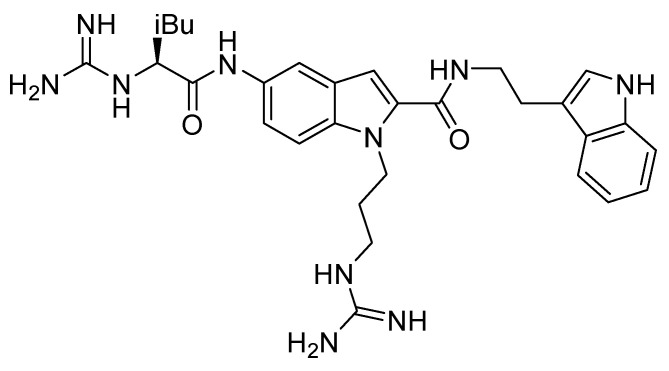
Structure of compound **10g**.

In line with the previously reported structural requirements, several derivatives were obtained; among them, compound **10g** (Figure 16) showed an IC_50_ of 3.0 μM in a competitive binding inhibition assay on human CXCR4-transfected Chinese hamster ovary (CHO) cells, using [125I]SDF-1 as a radioligand. In derivative **10g**, a 2-(3-indolyl)ethyl group was introduced at the position R2, while position R3 was decorated with a guanidinoacetyl group, further substituted at the α-carbon location with an isobutyl moiety. The chiral center introduced furnished further information about the best structural requirements for CXCR4 modulation; indeed, the activity assays suggested that S-isomers were more potent than the corresponding R-isomers [70].

### 4.5. Isothioureas Derivatives

**Figure 17:**
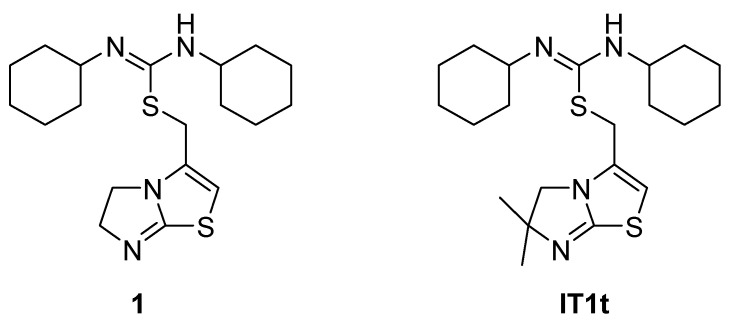
Structures of compound **1** and IT1t.

IT1t (Figure 17) is an orally available CXCR4 antagonist belonging to the chemical class of isothioureas. It was discovered in a screening of a Novartis compound collection against CXCR4 using a radioligand binding assay, leading to the identification of compound **1**, which was first described as a CXCR4 antagonist by Thoma et al. [74]. Compound **1** (Figure 17) was profiled in a series of in vitro assays, including radioligand binding assays. The membranes used were prepared from CEM cells, a T-lymphoblast cell line expressing human CXCR4, or from IR983F cells, a rat cell line expressing CXCR4. Compound **1** was able to inhibit calcium mobilization on both human and rat cells. It was also found to be selective for the CXCR4 receptor after being tested on a panel of receptors, such as CCR4, CCR5, CCR7, CCR9, and CXCR2. For these reasons, isothiourea was considered a promising scaffold and further developed, leading to IT1t [74].

IT1t, tested on Jurkat cells, was demonstrated to inhibit CXCR4/CXCL12 interaction. In particular, the cells were treated with the compound and then incubated with CXCL12^AF647^ (CXCL12 carrying an Alexa Fluor 647 moiety at its second-to-last amino acid position). The fluorescent signal of CXCL12^AF647^ was then quantified by flow cytometry; IT1t showed a dose-dependent inhibition of the CXCR4/CXCL12 interaction with IC_50_ values in the low nanomolar range (2.1 ± 0.37 nM). The antagonist activity of IT1t was further displayed by inhibition of the CXCL12-induced intracellular calcium flux (IC_50_ of 23.1 ± 4.6 nM) [75].

The crystal structures of CXCR4 bound to IT1t showed that the ligand occupies the pocket defined by side chains from helices I, II, III, and VII, making no contact with helices IV, V, and VI. The key interactions with the receptors have been identified as W94, D97, Y116, and E288.

**Figure 18:**
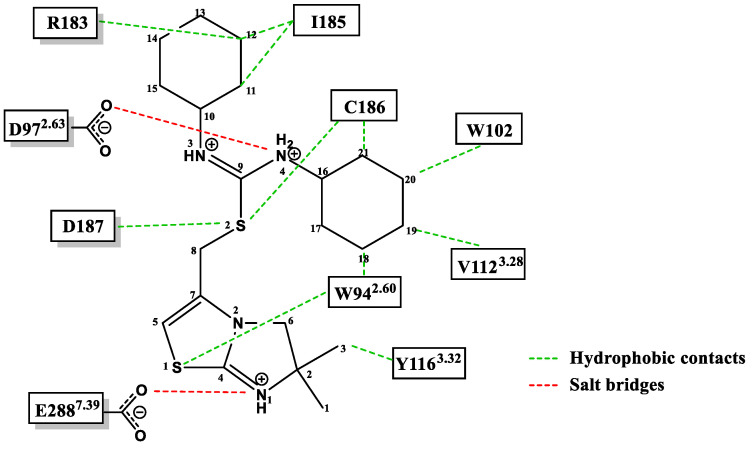
Interactions between CXCR4 and its ligand, IT1t. Adapted from Tulotta et al. [12].

The nitrogens of thiourea are both protonated, forming a salt bridge (2.7 Å) with the Asp97 ^2.63^ side chain. In fact, the methylation of one of the nitrogens reduced the binding affinity by about 100-fold [74]. Cyclohexane rings are located in small sub-pockets, making hydrophobic contact with the CXCR4 receptor. Then, the imidazo-thiazole ring has contact with helix VII, making a salt bridge (2.8 Å) between the protonated imidazo-thiazole N1 and Glu2887.39 [12] (Figure 18).

IT1t anticancer activity was evaluated on triple-negative breast cancer (TNBC), using a zebrafish xenotransplantation model expressing high levels of CXCR4. Cancer cells were pretreated with increasing concentrations of IT1t and incubated for 24 h, and the metabolic activity of the cells did not change during the 24 h incubation. Then, the engraftment of cells in the blood circulation of zebrafish embryos was performed, and pre-treatment in vitro caused a reduction in tumor burden at the secondary site in vivo, both at 2 and 4 days after injection [76].

### 4.6. Guanidine Derivatives

**Figure 19:**
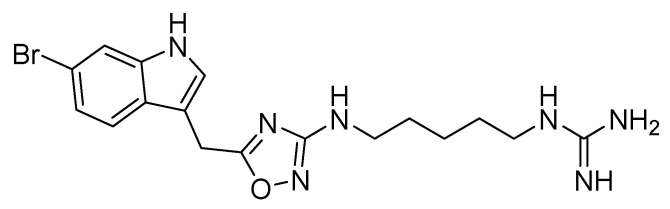
Structure of phidianidine A (PHIA).

Starting from a previously developed pharmacophoric model [77] for CXCR4 ligands, in which the essential anchoring points on CXCR4 consisted of an aromatic and a basic functional group, separated by a properly sized spacer, Vitale et al. applied a combined computational and experimental approach that led to the identification of phidianidine A (PHIA, Figure 19). PHIA is an alkaloid compound of marine origin, isolated from the marine ophisthobranch mollusk *Phidiana militaris* [78], and reported as a CXCR4 inhibitor with low micromolar activity.

**Figure 20:**
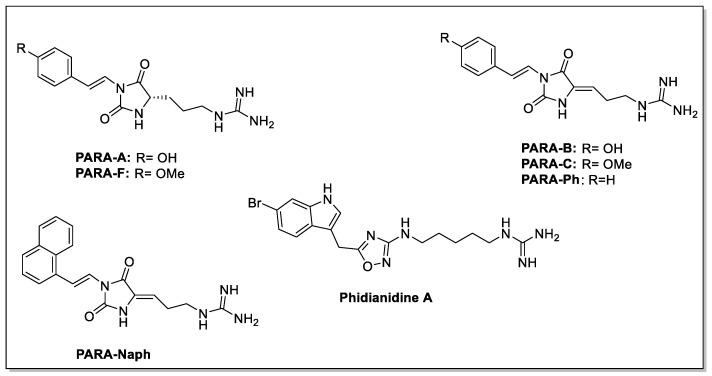
Structures of PARA-A, PARA-F, PARA-B, PARA-C, PARA-Ph, PARA-Naph, and phidianidine A.

To expand their ICB collection with natural molecules corresponding to the previously identified pharmacophoric model, Vitale’s research group identified the parazoanthine (hereafter dubbed as PARA) compound family [79]. They are hydantoin alkaloids isolated from the Mediterranean Sea anemone *Parazoanthus axinellae*; these compounds (Figure 20) share a strongly basic guanidium moiety separated from a (substituted) p-phenoxy group by a nine-bond spacer including a hydantoin ring. PARAs differ from each other either in the substitution pattern of the p-phenoxy group, or in the saturation state of the 5,6 extracyclic bond on the hydantoin ring.

The synthesized derivatives were evaluated for their ability to inhibit proliferation, migration, and CXCL12-dependent ERK1/2 MAP kinase phosphorylation/activation in GH4C1 cells of the rat pituitary adenoma cell line; although all compounds were able to antagonize the effects of CXCL12, with varying potency and efficacy, parazoanthine-B (Figure 20) was identified as the most potent CXCR4 antagonist, with an IC_50_ value of 9.3 nM.

**Figure 21:**
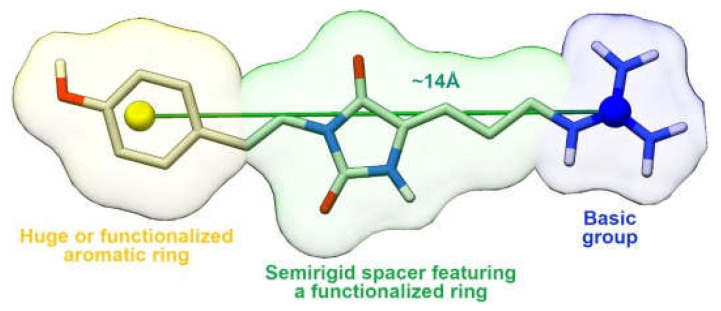
Optimized pharmacophoric mode. Adapted from Vitale et al. [79].

These results allowed the development of a more stringent pharmacophoric model (Figure 21); in fact, by comparing the scaffolds of phidianidine A and parazoanthines B-C and considering the higher inhibitory potency of PARA derivatives, it was assumed that a shorter distance (~14 Å instead of 18 Å) between the guanidine group and the aromatic moiety improves activity. Probably, the shorter linker and the two double bonds on the alkyl chain reduce the flexibility of the ligands, causing a reduction in the unfavorable entropy contribution upon binding. Furthermore, it is evident that the presence of a voluminous aromatic group is not mandatory for the antagonism; however, the phenyl group must compensate for its small size with a hydroxyl or methoxyl group to create H-bonds with polar residues in the sub-pocket housing the aromatic part. Finally, in both fidianidine A and parazoanthines, the alkyl linker has a five-membered ring capable of forming H-bonds with the receptor.

The identification of a new pharmacophoric model based on the hydantoin scaffold supports this class of small molecules as potent CXCR4 antagonists in tumoral pathologies characterized by an overexpression of this receptor.

The Table 1 summarizes the structures and the scaffolds reported in the sections above.

## 5. Conclusions

In the last years, the influence of the CXCR4 axis in tumor pathogenesis has become increasingly relevant. The interaction between cancer cells and their microenvironment, which preserves malignant cells from genotoxic stresses like chemotherapy, represents a promising target for enhancing anti-cancer therapies. CXCR4 and CXCL12, which are expressed on both tumor cells and surrounding cells, play a crucial role in the communication between tumor cells and their microenvironment: this explains the importance of identifying new molecules potentially able to inhibit these receptors and thus block tumor proliferation. This review summarizes the most important mechanisms involving the CXCR4/CXCL12 axis, focusing particularly on the processes of proliferation, angiogenesis, and metastasis, and demonstrating the extensive involvement of this axis in the processes described. Therefore, a series of CXCR4/CXCL12 axis antagonists that may be further developed as antitumor agents were described, and the key molecular motifs in the interactions with the receptors were highlighted. In fact, although the importance of CXCR4 in cancer is widely demonstrated, currently no small molecules are used for this purpose in clinical practice. Therefore, the optimization of existing scaffolds or the discovery of new ones, based on SAR studies, may pave the way for new CXCR4 antagonists useful in tackling multiple types of cancer.

## Figures and Tables

**Table 1:** Small molecules targeting CXCR4/CXCL12 axis.

Name	Structure	Mechanism	Application
**AMD3100**	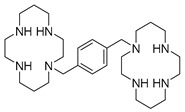	Mobilization of hematopoietic stem cells.	Currently used to prepare patients for stem cell transplant.
**AMD3100 derivative MSX-122**	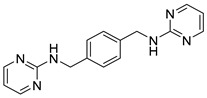	Inhibition of metastasis and inflammation.	Withdrawn from clinical trials for its toxicity
**Amide-based scaffold CPZ1344**	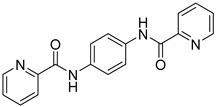	Anticancer effects on glioma cell lines.	Under investigation.
**Amide–sulfonamide derivatives**	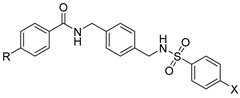	Block of CXCR4^+^ cancer cell invasion.	Under investigation.
**Tetrahydroquinoline–benzimidazole-based scaffold** **AMD11070**	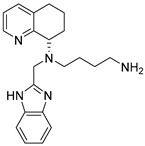	Inhibition migration of the melanoma cell line A375.	Approved by FDA in WHIM syndrome.
**Indole derivative 10g**	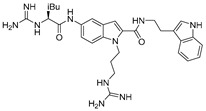	CXCR4 antagonist.	Under investigation.
**IT1t**	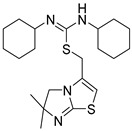	Inhibition of CXCR4/CXCL12 interaction.	Under investigation on triple-negative breast cancer.
**Guanidine-based derivative** **PHIA**	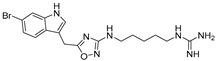	Inhibition of proliferation and CXCL12-dependent migration in GH4C1 cells of the rat pituitary adenoma cell line.	Under investigation.

## Data Availability

Data sharing is not applicable to this article.
